# Oscillometry in Lung Function Assessment: A Comprehensive Review of Current Insights and Challenges

**DOI:** 10.7759/cureus.47935

**Published:** 2023-10-29

**Authors:** Souvik Sarkar, Ulhas Jadhav, Babaji Ghewade, Syamal Sarkar, Pankaj Wagh

**Affiliations:** 1 Respiratory Medicine, Jawaharlal Nehru Medical College, Datta Meghe Institute of Higher Education & Research, Wardha, IND; 2 Respiratory Medicine, Advanced Chest Care Centre, Ranchi, IND

**Keywords:** pulmonary function test, pediatric care, non-invasive, respiratory diseases, lung function assessment, oscillometry

## Abstract

Oscillometry, a non-invasive technique for assessing lung function, has gained significant recognition and importance in modern pulmonary medicine. This comprehensive review thoroughly explores its principles, applications, advantages, limitations, recent innovations, and future directions. Oscillometry's primary strength lies in its ability to offer a holistic assessment of lung mechanics. Unlike traditional spirometry, oscillometry captures the natural airflow during quiet breathing, making it suitable for patients of all ages and those with severe respiratory conditions. It provides a comprehensive evaluation of airway resistance, reactance, and compliance, offering insights into lung function that were previously challenging to obtain. In clinical practice, oscillometry finds extensive application in diagnosing and managing respiratory diseases. It plays a pivotal role in asthma, chronic obstructive pulmonary disease (COPD), and interstitial lung diseases. By detecting subtle changes in lung function before symptoms manifest, oscillometry facilitates early interventions, improving disease management and patient outcomes. Oscillometry's non-invasive and patient-friendly nature is precious in pediatric care, where traditional spirometry may be challenging for young patients. It aids in diagnosing and monitoring pediatric respiratory disorders, ensuring that children receive the care they need from an early age. Despite its many advantages, oscillometry faces challenges, such as the need for standardized protocols and the complexity of data interpretation. However, ongoing efforts to establish global standards and provide education and training for healthcare professionals aim to address these issues. Looking ahead, oscillometry holds great promise in the field of personalized medicine. With its ability to tailor treatment plans based on individualized lung function data, healthcare providers can optimize therapy selection and dosing, ultimately improving patient care and quality of life. In conclusion, oscillometry is poised to play an increasingly pivotal role in modern pulmonary medicine. As standardization efforts continue and technology evolves, it is an indispensable tool in the clinician's arsenal for diagnosing, managing, and personalizing respiratory care, ultimately leading to improved patient outcomes and better respiratory health.

## Introduction and background

Lung function assessment plays a pivotal role in pulmonary medicine, enabling the measurement and evaluation of various parameters related to the respiratory system's functionality. This assessment is essential for diagnosing and managing respiratory diseases, monitoring treatment effectiveness, and maintaining overall lung health. At its core, the primary objective of lung function assessment is to provide healthcare professionals with valuable insights into the functionality of the lungs, facilitating informed decisions concerning patient care [1-3].

The primary parameters examined in lung function testing encompass lung capacity, airflow, gas exchange, and airway resistance. These measurements are critical for the diagnosis of conditions such as asthma, chronic obstructive pulmonary disease (COPD), interstitial lung diseases, and other respiratory disorders. Moreover, lung function assessment plays a pivotal role in understanding disease progression, predicting outcomes, and tailoring individualized treatment plans [4].

Among the most significant advancements in lung function assessment is the utilization of oscillometry. Oscillometry, also known as impulse oscillometry, is a non-invasive technique that delves into the mechanical properties of the respiratory system. It does so by analyzing pressure and flow signals generated during spontaneous breathing. Unlike traditional spirometry, which relies on forced expiratory maneuvers, oscillometry offers a more comprehensive and nuanced assessment of lung function [5].

The significance of oscillometry lies in its capacity to provide invaluable insights into the peripheral airways, a facet often overlooked by conventional lung function tests. It furnishes information regarding airway resistance, reactance, and compliance, thereby enabling healthcare professionals to detect abnormalities in small airways and the lung periphery. This capability proves particularly beneficial in the early diagnosis and monitoring of diseases characterized by peripheral airway involvement, such as asthma and specific forms of COPD [6].

This comprehensive review endeavors to explore oscillometry in lung function assessment in detail, elucidating its underlying principles, applications, advantages, limitations, and current developments. Our aim is to shed light on how oscillometry has revolutionized the landscape of respiratory medicine and its potential to transform patient care. To this end, we will explicitly outline the purpose of this review, including the specific knowledge gaps we intend to address and the anticipated contributions to the field of respiratory medicine.

## Review

Historical perspective

Early Methods for Lung Function Assessment

Percussion (18th and 19th centuries): Physicians in the 18th and 19th centuries employed percussion as a diagnostic technique for assessing lung function. This method involved tapping the patient's chest and listening for changes in sound, which could indicate the presence of conditions such as pneumonia or pleural effusion. The percussion was a valuable tool in its time, providing insights into the density and composition of the underlying lung tissue and chest cavity [7].

Auscultation (early 19th century): René Laennec, a pioneering physician of the early 19th century, introduced the stethoscope, a transformative innovation in medical diagnostics. Auscultation, or listening to breath sounds, became possible with the stethoscope, allowing physicians to detect abnormalities in lung sounds and diagnose various lung diseases. Laennec's invention marked a significant advancement in respiratory medicine and significantly improved the accuracy of lung function assessment [8].

In the 19th century, a significant milestone in the history of lung function assessment was marked by the development of rudimentary spirometers. Although less advanced than contemporary technology, these early spirometers played a vital role in shaping the trajectory of lung function testing. Spirometry, at its core, involves the measurement of the volume of air expelled from the lungs during forced expiration. This innovation represented a fundamental shift in how physicians could evaluate respiratory health. These early spirometers allowed for quantifying crucial lung volumes and capacities, providing healthcare professionals with a means to assess vital parameters, including forced vital capacity (FVC) and forced expiratory volume in one second (FEV1). The significance of this development cannot be overstated, as it laid the foundational groundwork for more sophisticated and precise lung function testing methods that would emerge in the following decades [9]. By quantifying these key parameters, spirometry not only provided a window into the functioning of the lungs but also offered valuable insights into diagnosing and managing respiratory diseases. This innovation opened doors to a more comprehensive understanding of lung health and marked a pivotal step forward in the evolution of respiratory medicine.

Emergence of Oscillometry in Pulmonary Medicine

The emergence of oscillometry as a distinct method for lung function assessment can be traced back to the mid-20th century. It gained recognition as an alternative to traditional spirometry, offering unique advantages in evaluating respiratory mechanics. One of oscillometry's key innovations was its ability to assess lung function without requiring forced expiratory maneuvers, making it suitable for a broader range of patients, including children and individuals with severe respiratory conditions [10]. While the initial adoption of oscillometry in clinical practice could have been faster, this was primarily hindered by the lack of standardized techniques and limited access to appropriate equipment. However, as the technology matured and its clinical utility became more evident, oscillometry began to gain traction among pulmonary specialists [11].

Milestones in the Development of Oscillometry Techniques

Introduction of commercial oscillometers (1970s): The first commercial oscillometers were introduced in the 1970s, marking a pivotal moment in lung function assessment. These early devices enabled healthcare providers to measure respiratory impedance, providing valuable insights into airway resistance and reactance. This development made oscillometry accessible for clinical use and laid the foundation for its subsequent advancements [12].

Advancements in technology: Oscillometry technology underwent continuous refinement over the decades. Advances in hardware and software have significantly improved the accuracy and precision of measurements. These innovations allowed clinicians to assess the mechanical properties of the entire respiratory system, providing a more comprehensive view of lung function. Enhanced signal processing algorithms, improved device design, and increased measurement frequencies contributed to the refinement of oscillometry techniques [13].

Oscillometry's credibility and clinical relevance have been rigorously substantiated through extensive research and validation studies. These validation efforts were designed not only to establish the reliability of oscillometry measurements but also to illustrate their precision in accurately diagnosing a wide spectrum of respiratory disorders. These studies played a pivotal role in confirming the utility of oscillometry in conditions as diverse as asthma, chronic obstructive pulmonary disease (COPD), and interstitial lung diseases [14].

The culmination of these validation studies provides compelling evidence of the effectiveness and trustworthiness of oscillometry as a diagnostic tool in clinical practice. In the context of asthma, the validation studies have consistently shown that oscillometry can accurately detect airway resistance and reactance, aiding in diagnosing and monitoring this prevalent respiratory condition. Similarly, in the case of COPD, the validation studies have demonstrated the utility of oscillometry in detecting airway abnormalities, providing clinicians with valuable insights into disease progression and treatment responses. Moreover, for interstitial lung diseases, the validation studies have highlighted the ability of oscillometry to assess lung function changes associated with these conditions, contributing to early diagnosis and tailored treatment strategies [14].

These validation efforts have essentially solidified oscillometry's role as a dependable and valuable tool in respiratory medicine. This body of evidence has paved the way for integrating oscillometry into routine clinical practice, underscoring its importance in enhancing the accuracy and comprehensiveness of lung function assessment in patients with a diverse range of respiratory disorders [14].

Integration into clinical practice: Oscillometry gradually found its place in clinical practice guidelines and recommendations for lung function assessment. It became recognized as an essential complement to traditional spirometry, especially in cases where small airway abnormalities or peripheral lung diseases were suspected. Oscillometry's ability to provide insights into airway mechanics and peripheral lung function makes it a valuable tool for diagnosing and monitoring various respiratory conditions. As a result, clinicians increasingly incorporated oscillometry into their routine assessments, further solidifying its role in modern pulmonary medicine [15].

Principles of oscillometry

Basics of Oscillation and Impedance

Oscillation: Oscillation, within the context of lung function assessment, refers to the rhythmic movement of air in and out of the respiratory system during natural, spontaneous breathing. Unlike spirometry, which involves forced maneuvers and deep inhalations, oscillometry captures the natural oscillatory flow of air as a patient breathes quietly and comfortably. This non-invasive approach is particularly advantageous because it eliminates the need for patient cooperation and maximal effort. Oscillation-based measurements are well-suited for individuals of all ages and conditions, including those with limited lung function or severe respiratory conditions. The gentle and effortless nature of oscillometry testing enhances patient comfort and minimizes discomfort, making it an accessible and versatile tool in respiratory medicine [16].

Impedance: In its broader electrical context, impedance signifies the resistance to the flow of alternating current in a circuit. In the specific domain of oscillometry, respiratory impedance refers to the opposition encountered by the oscillatory airflow within the respiratory system. Various mechanical properties influence this impedance, including the airways' resistance, the lung tissue's reactance, and the chest wall's compliance. By measuring respiratory impedance, oscillometry provides valuable information about the intricate mechanics of the respiratory system. It allows clinicians to assess how airflow is impeded and redirected as it travels through the airways, providing insights into airway resistance, tissue compliance, and other factors that affect lung function [12].

Measurement of Respiratory Impedance

The measurement of respiratory impedance through oscillometry is a valuable technique that provides insight into the dynamics of lung function. This process begins with generating oscillations within the respiratory system, typically through an oscillatory source such as a loudspeaker. These oscillations are introduced into the patient's airway as they breathe naturally. Critical sensors placed at the airway opening play a pivotal role in capturing the intricate variations in pressure and airflow resulting from these oscillations during spontaneous breathing [17].

The recorded pressure and flow signals are then subjected to rigorous analysis, yielding respiratory impedance. This impedance is a complex value comprising two essential components: resistance (R) and reactance (X). Resistance represents the opposition to airflow within the respiratory system, primarily reflecting the caliber and patency of the airways. It quantifies the real part of the impedance and is measured in units such as cmH2O/L/s. An increase in resistance suggests that airflow encounters greater resistance, often indicative of narrowed or constricted airways [18].

In contrast, reactance reflects the elastic and inertial properties of the respiratory system. It offers insights into lung compliance-the ability of the lungs to expand and contract and the capacity to store and release energy during the breathing cycle. Reactance is quantified as the imaginary part of the impedance, measured in units like cmH2O/L. A decrease in reactance may signify decreased lung compliance, while an increase could indicate increased lung compliance or other changes in the elastic properties of lung tissue [19].

Oscillometry's ability to derive these complex impedance values empowers clinicians with a more nuanced understanding of lung function. Healthcare providers gain insights into airway resistance, lung compliance, and other critical parameters essential for diagnosing and monitoring various respiratory conditions by assessing resistance and reactance. This non-invasive technique enhances lung mechanics assessment and contributes to a deeper comprehension of the complex interplay between the respiratory system's resistive and reactive components, ultimately improving patient care and treatment decision-making [20].

Interpretation of Oscillometry Data

Airway resistance (R): Airway resistance, represented by the resistance (R) value, provides information about the ease of airflow through the respiratory system. An increase in airway resistance suggests obstructive conditions, such as asthma or chronic bronchitis. Elevated resistance values often indicate narrowing or constriction of the airways, which results in increased airflow resistance. Monitoring changes in airway resistance can be instrumental in diagnosing and managing these obstructive respiratory conditions [21].

Reactance (X): It reflects the elastic properties and compliance of the lung tissue. A decrease in reactance suggests decreased lung compliance, which may be seen in conditions like pulmonary fibrosis. Reduced lung compliance means the lung tissue is less able to expand and contract, leading to stiffness. Conversely, an increase in reactance may indicate increased lung compliance, as seen in conditions like emphysema, where the lung tissue is overly elastic and less able to recoil efficiently [22].

Frequency dependence: Oscillometry measures impedance at various frequencies. Analyzing how impedance changes with frequency can provide additional diagnostic information. For example, resistance tends to increase more significantly in asthma at higher frequencies, a phenomenon known as "frequency dependence." This characteristic is due to the constriction of smaller airways, which are more affected by higher-frequency oscillations. Frequency-dependent changes in impedance can be a valuable marker for diagnosing and monitoring asthma [23].

Comparison with normative data: To interpret oscillometry data accurately, clinicians often compare the obtained measurements with normative values specific to the patient's age, gender, and height. This comparison helps determine whether the measured parameters fall within the expected ranges for the patient's demographic group. Deviations from normative values can indicate abnormal lung function and guide further evaluation and intervention [24].

Monitoring over time: Oscillometry's utility extends beyond diagnosis; it is also valuable for monitoring disease progression and treatment efficacy. Serial impedance measurements allow clinicians to assess changes in lung function over time. By tracking trends in airway resistance, reactance, and other parameters, healthcare providers can gauge the effectiveness of treatment interventions and make timely adjustments to optimize patient care. This longitudinal approach enhances the precision of disease management [12].

Clinical applications of oscillometry

Diagnosis and Monitoring of Respiratory Diseases

Oscillometry is an indispensable tool in respiratory medicine, offering critical support in diagnosing and monitoring various respiratory diseases. Three primary respiratory conditions where oscillometry plays a pivotal role are asthma, chronic obstructive pulmonary disease (COPD), and interstitial lung diseases (ILDs) [11].

In the case of asthma, a complex chronic respiratory disorder characterized by airway inflammation and bronchoconstriction, oscillometry provides invaluable insights. First, it aids in assessing airway hyperresponsiveness, a hallmark of asthma. Oscillometry can detect increased airway resistance and reduced reactance, serving as sensitive indicators of asthma exacerbations. Furthermore, oscillometry's ability to identify early airflow limitations is particularly noteworthy. Even before clinical symptoms manifest, oscillometry can detect subtle changes in airway function, enabling prompt intervention and proactive asthma management. Additionally, oscillometry facilitates the monitoring of disease severity over time and tracks the response to bronchodilator therapy. This continuous assessment empowers healthcare providers to adjust treatment regimens based on objective data, ensuring patients receive optimal care tailored to their asthma characteristics [25].

In individuals with COPD, a progressive respiratory condition often characterized by small airway abnormalities, oscillometry comprehensively evaluates lung function. It is crucial in assessing airway resistance and reactance changes associated with COPD exacerbations. By detecting these changes early, oscillometry helps identify exacerbations before they escalate, guiding timely interventions to improve patient outcomes. Furthermore, oscillometry is a valuable tool for monitoring disease progression in COPD patients. It identifies individuals at risk of exacerbations and enables healthcare providers to make informed therapeutic decisions to optimize bronchodilator therapy. This data-driven approach enhances the overall management of COPD, ultimately improving the quality of life for affected individuals [26].

In interstitial lung diseases (ILDs), a diverse group of disorders affecting the lung's interstitium, oscillometry offers critical diagnostic and monitoring capabilities. Oscillometry is instrumental in detecting changes in lung compliance and reactance associated with fibrotic ILDs. These changes indicate the impact of ILDs on lung function and provide valuable insights for healthcare providers. By assessing these parameters, oscillometry aids in evaluating the progression of ILDs and guides therapeutic interventions. Adjusting treatment strategies based on oscillometry data enables healthcare providers to provide targeted care for individuals with ILDs, enhancing their quality of life and potentially slowing disease progression [27].

Assessment of Airway Resistance

Detecting peripheral airway abnormalities: Oscillometry excels at identifying abnormalities in the small, peripheral airways of the lungs, which are often overlooked by traditional spirometry. These peripheral airways are crucial in conditions like asthma and early-stage chronic obstructive pulmonary disease (COPD), where small airway involvement is common. Oscillometry's ability to capture data from these areas provides a more comprehensive evaluation of lung function and helps detect subtle abnormalities that might go unnoticed [28].

Quantifying changes in resistance: Oscillometry provides quantitative measurements of airway resistance. This capability allows clinicians to monitor changes in resistance over time, enabling them to assess the effectiveness of treatment interventions. Quantitative data precisely track improvements or deteriorations in airway function, facilitating data-driven clinical decisions and adjustments to treatment plans [29].

Differentiating between obstructive and restrictive patterns: Oscillometry analysis, which includes resistance and reactance measurements, can assist in distinguishing between obstructive and restrictive lung diseases. The distinct patterns of resistance and reactance data provide valuable diagnostic insights. For example, oscillometry can help differentiate obstructive conditions like asthma and COPD, characterized by increased resistance, from restrictive conditions like pulmonary fibrosis, which exhibit specific patterns of reactance. This differentiation is essential for accurate diagnosis and appropriate treatment strategies [10].

Pediatric Applications

Diagnosis of pediatric respiratory conditions: Oscillometry is critical in diagnosing and monitoring pediatric respiratory disorders. It is instrumental in asthma, bronchiolitis, and cystic fibrosis conditions. Oscillometry provides objective data on airway resistance, reactance, and compliance, which can aid in confirming diagnoses and assessing disease severity. Its non-invasive nature and minimal patient effort make it well-suited for use in pediatric populations, even in young children with difficulty performing spirometry or other pulmonary function tests [30].

Assessment of lung growth and development: Oscillometry can track lung growth and development in children over time. This longitudinal assessment helps healthcare providers identify abnormalities or delays in lung function maturation. Monitoring lung growth is crucial for identifying respiratory conditions that may manifest or worsen as a child grows, such as congenital lung abnormalities or developmental lung diseases. Oscillometry provides valuable insights into these processes and allows for early intervention when necessary [31].

Monitoring and adjusting pediatric treatment: Oscillometry enables clinicians to monitor the effectiveness of treatment regimens for pediatric patients with respiratory conditions. By regularly assessing lung function with oscillometry, healthcare providers can track changes in airway resistance and other parameters, allowing for the timely adjustment of therapy plans. This personalized approach ensures that children receive optimal care, with treatment regimens tailored to their evolving lung function characteristics. It also aids in preventing exacerbations and optimizing disease management in pediatric respiratory conditions [30].

Monitoring Response to Treatment

Treatment optimization: Clinicians can utilize oscillometry to optimize medication regimens for individual patients. Healthcare providers can fine-tune therapies to match the specific characteristics of a patient's respiratory condition by assessing changes in lung function parameters, such as airway resistance and reactance. This tailored approach ensures that patients receive the most effective treatments, potentially leading to improved symptom management and overall quality of life [32].

Tracking changes over time: Serial oscillometry measurements provide valuable insights into how a patient's lung function evolves. This longitudinal assessment allows healthcare providers to monitor disease progression and the impact of treatment interventions. It helps evaluate whether the current treatment plan effectively stabilizes or improves lung function or if adjustments are needed to manage the patient's condition [33].

Early detection of treatment failures: Oscillometry can detect treatment failures or suboptimal responses earlier than conventional clinical measures. Subtle changes in lung function that may not be immediately apparent through symptom assessment or spirometry can be detected by oscillometry. This early detection capability enables healthcare providers to intervene promptly, adjusting treatment plans to enhance efficacy and prevent disease exacerbations or complications [34].

Advantages and limitations

Advantages of Oscillometry Compared to Conventional Spirometry

Non-invasiveness: Oscillometry is a non-invasive technique requiring minimal patient effort. Unlike spirometry, which involves forced breathing maneuvers, oscillometry captures lung function during natural breathing. This characteristic makes it suitable for many patients, including children, the elderly, and individuals with severe respiratory conditions. It reduces the discomfort and potential complications of invasive tests [35].

Comprehensive assessment: Oscillometry provides a comprehensive assessment of lung function by analyzing airway resistance, reactance, and compliance. This multi-dimensional approach allows for detecting abnormalities in the peripheral airways, offering a more nuanced evaluation of lung mechanics. It provides a more detailed picture of respiratory health than traditional spirometry can achieve [36].

Early detection: Oscillometry can detect subtle changes in lung function before they become clinically apparent. This early detection is particularly valuable in managing respiratory conditions such as asthma and chronic obstructive pulmonary disease (COPD), where timely intervention can prevent exacerbations and improve long-term outcomes. It enhances identifying disease progression and implementing proactive measures [37].

Serial monitoring: Oscillometry enables serial monitoring of lung function over time. Clinicians can use oscillometry to track changes in lung function and assess the effectiveness of treatment regimens. Real-time data allows for the adjustment of therapies based on individual patient responses. This dynamic monitoring enhances the precision of patient care [23].

Oscillometry is an excellent choice for assessing lung function in pediatric patients, offering particular advantages when dealing with younger individuals who may encounter challenges in performing spirometry correctly due to age or cooperation limitations. However, it is crucial to delve into the nuances associated with its application in this population, recognizing that pediatric care often necessitates a specialized approach [38]. Oscillometry's versatility becomes evident when we consider its ability to adapt to the unique requirements of pediatric patients. It caters to children of various ages, making it a valuable tool for diagnosing and managing pediatric respiratory conditions. Moreover, it significantly contributes to early intervention and improved care for young patients [38].

In pediatric practice, certain age-related differences and cooperation limitations may impact the application of oscillometry. Young children, in particular, may find it challenging to follow instructions or maintain consistent breathing patterns during the test. This may lead to variations in results and necessitate additional considerations when interpreting the data. To address these nuances, it becomes essential for healthcare professionals to employ age-appropriate techniques, engage in patient education and preparation, and employ specialized pediatric-specific equipment when necessary [38]. Furthermore, certain pediatric respiratory conditions may present unique challenges when utilizing oscillometry. The review will explore these condition-specific considerations, offering insights into how oscillometry can be adapted to meet the needs of young patients with various respiratory disorders.

No need for specialized training: While interpreting oscillometry data requires expertise, the test does not require specialized patient training. Patients can easily undergo oscillometry without complex maneuvers or extensive training. This reduces the potential for errors related to patient technique, enhancing the reliability of results [35].

Limitations and Challenges in Oscillometry

Complex interpretation: Oscillometry data can be more complex than traditional spirometry results. Extracting meaningful insights from impedance measurements requires specialized knowledge and training. Clinicians may need to invest additional time and effort in understanding oscillometry principles and data interpretation, which can hinder its widespread adoption [6].

One of the central challenges in oscillometry is the lack of standardized protocols and reference values. This absence of uniformity can make comparing results obtained from different laboratories or devices considerably more difficult. While ongoing efforts are directed towards establishing universal guidelines and reference values for oscillometry, it is important to acknowledge that, until such standardization is accomplished, variations in testing methodologies may continue to persist [39].

Cost and equipment: High-quality oscillometers can be expensive, limiting their accessibility, particularly in resource-constrained healthcare facilities. The cost of equipment, as well as the availability of oscillometry devices, can vary by region. These factors can hinder the widespread use of oscillometry in some healthcare settings [12].

Limited normative data: Normative data for oscillometry may be limited, especially in specific populations like pediatric patients. This limitation can make determining what constitutes regular or abnormal values challenging, particularly in age groups with scarce reference data. Further research and data collection are needed to establish robust normative values for various patient populations [40].

Patient factors: While oscillometry is less effort-intensive than spirometry, patient-related factors such as cooperation, posture, and breathing patterns can still influence the results. Standardizing these factors across different patients can be challenging, and healthcare providers must ensure that patients are adequately trained and instructed to obtain reliable oscillometry measurements [26].

Potential Pitfalls in Interpretation

Inadequate calibration: To ensure the accuracy of measurements, it is of utmost importance to properly calibrate oscillometry equipment. Adequate calibration guarantees that the equipment functions correctly and yields reliable and consistent oscillometry measurements. Clinicians and healthcare providers should adhere to manufacturer recommendations for calibration procedures and conduct regular verifications to confirm the equipment's proper functioning. By dedicating attention to calibration procedures, healthcare professionals can safeguard against erroneous results and maintain the integrity of the data acquired [41].

Variability: Oscillometry measurements can exhibit variability, and multiple readings may be necessary to obtain a reliable assessment of lung function. This variability can be influenced by patient cooperation, breathing patterns, and even the time of day. Clinicians should be aware of the potential for variability and consider taking multiple measurements, especially when assessing patients with fluctuating or complex respiratory conditions. Averaging multiple readings can help mitigate the impact of variability on interpretation [42].

Clinical correlation: While oscillometry provides valuable insights into lung function, it is essential to interpret the data in the context of the patient's clinical history and presentation. Abnormal oscillometry results should prompt further evaluation to confirm the diagnosis and assess the clinical significance of the findings. Clinicians should use oscillometry as one tool within the broader diagnostic framework and consider it alongside other clinical data and test results [43].

Educational needs: Both healthcare providers and patients may require education and training to understand the value and limitations of oscillometry. Clinicians should take the initiative to inform patients about the purpose of oscillometry, its role in their healthcare, and what the results signify. Ensuring that users are adequately informed can enhance patient cooperation, adherence to testing protocols, and the overall quality of care. Similarly, ongoing education for healthcare providers is essential to update them on the best oscillometry interpretation practices [44].

Current insights and innovations

Recent Advances in Oscillometry Technology

Portable devices: The development of smaller, more portable oscillometers has revolutionized lung function assessment. These compact devices are easier to transport and use in various healthcare settings, including primary care clinics, and even for home-based monitoring in select patient populations. Portable oscillometers increase accessibility to lung function assessment and facilitate more frequent monitoring, especially for patients with chronic respiratory conditions [45].

Advanced signal processing: Oscillometry devices now employ advanced signal processing algorithms that improve the accuracy and reliability of measurements. These algorithms enhance the ability to isolate specific respiratory impedance components, such as airway resistance and reactance, making it easier for clinicians to interpret results accurately. The precision offered by advanced signal processing contributes to more confident clinical decision-making [46].

Integration with smart devices: Modern oscillometers often include features enabling smartphone or tablet connectivity. This integration allows for real-time data analysis, remote monitoring, and seamless data sharing between patients and healthcare providers. Patients can engage with their lung function data through user-friendly apps, and telehealth consultations become more effective with immediate access to oscillometry results. This connectivity fosters patient engagement and empowers individuals to actively manage their respiratory health [47].

Multi-frequency analysis: A notable feature of many modern oscillometry devices is their ability to perform multi-frequency analysis. This innovation allows for a comprehensive assessment of respiratory mechanics by examining impedance at various frequencies. Clinicians can utilize this capability to gain insights into airway resistance and reactance across different lung regions, offering a more detailed evaluation of lung function. Multi-frequency analysis proves invaluable for detecting subtle abnormalities and monitoring disease progression with enhanced precision [48].

Pediatric-focused features: Innovations in pediatric oscillometry cater to the unique needs of young patients. User-friendly interfaces and interactive games designed to keep children engaged during testing make the experience more child friendly. Additionally, these devices incorporate reference data specific to pediatric age groups, ensuring that oscillometry results are interpreted in the context of normal values relevant to children's development. This pediatric-focused approach facilitates early diagnosis and improved monitoring of pediatric respiratory conditions [49].

Integration With Other Diagnostic Tools

Imaging techniques: Combining oscillometry with advanced imaging modalities such as computed tomography (CT) scans or magnetic resonance imaging (MRI) represents a powerful approach to respiratory assessment. This integration allows clinicians to correlate structural changes in the lung with functional abnormalities detected by oscillometry. Healthcare providers gain a more comprehensive understanding of respiratory conditions by fusing data from multiple sources. For example, this approach can reveal how specific lung pathologies relate to changes in airway resistance, offering valuable insights for diagnosis and treatment planning [50].

Biomarkers and inflammatory markers: Researchers are exploring the integration of oscillometry with biomarker measurements, such as exhaled breath markers and blood tests, to identify molecular and cellular markers associated with respiratory diseases. This multi-modal approach enables the identification of specific biological indicators that can be early signs of disease or can reflect disease severity. By combining biomarker data with oscillometry results, healthcare providers can enhance their diagnostic accuracy, monitor disease progression, and personalize treatment strategies based on these molecular insights [51].

Artificial intelligence (AI) and machine learning: AI algorithms can analyze oscillometry data alongside other clinical and imaging data, enabling a more holistic approach to respiratory assessment. By leveraging AI and machine learning, healthcare providers can predict disease progression, treatment responses, and patient outcomes more precisely. These data-driven approaches can revolutionize personalized medicine in respiratory care, allowing treatment plans tailored to each patient's unique lung function characteristics, genetics, imaging findings, and clinical history [52].

Emerging Trends in Research

Precision medicine: Researchers are exploring the integration of oscillometry with genetic and molecular profiling to advance the concept of precision medicine in respiratory care. By combining genetic information and oscillometry data, clinicians can tailor treatment plans to individual patients, optimizing therapy selection and dosing for better outcomes. This personalized approach can enhance treatment efficacy and significantly minimize adverse effects [53].

Longitudinal studies: Long-term oscillometry monitoring is gaining prominence, particularly in managing chronic respiratory diseases. Researchers are conducting comprehensive longitudinal studies to gain insights into disease trajectories, identify early predictors of exacerbations, and refine management strategies. Healthcare providers can make informed decisions about disease progression and treatment adjustments by analyzing oscillometry data over extended periods [54].

Artificial intelligence in diagnosis: Applying artificial intelligence (AI) and machine learning to oscillometry data analysis is an emerging trend. Researchers are training AI algorithms to recognize patterns and correlations in oscillometry data, enabling automated diagnosis or risk prediction. This innovative approach has the potential to expedite the diagnostic process, particularly in cases of complex respiratory conditions, and improve overall disease management through timely interventions [55].

Telemedicine and remote monitoring: The growing adoption of telemedicine has spurred research into remote oscillometry monitoring for patients with chronic respiratory conditions. Remote oscillometry allows healthcare providers to track patients' lung function over time, offering real-time data on their respiratory health. This data can be instrumental in identifying early signs of deterioration, enabling healthcare professionals to intervene promptly and optimize patient care, even from a distance [56].

Pediatric respiratory research: Ongoing studies are dedicated to refining oscillometry techniques for pediatric populations, including infants and young children. Pediatric respiratory research aims to improve the accuracy of oscillometry assessments in children and enhance early detection of respiratory disorders. This is particularly critical, as pediatric respiratory conditions often require early diagnosis and intervention to ensure optimal long-term outcomes [57].

Challenges and future directions

Standardization and Consistency in Oscillometry Measurements

Lack of standardization: One of the fundamental hurdles in oscillometry is the need for standardized protocols and reference values. This variability in testing methodologies and interpretation can hinder accurate assessment and diagnosis. To address this challenge, future directions should prioritize the establishment of comprehensive, evidence-based standards for oscillometry. These standards should encompass every aspect of oscillometry, from device calibration to testing procedures and data interpretation. Developing a unified set of guidelines will help mitigate discrepancies and enhance the consistency of oscillometry measurements, regardless of where they are performed [58].

Global efforts: Collaboration among healthcare organizations, researchers, and regulatory bodies on a global scale is paramount for advancing the standardization of oscillometry. Future directions should emphasize international partnerships to establish universal standards for oscillometry. By leveraging collective expertise and resources, these collaborative efforts can create a framework ensuring uniformity in worldwide oscillometry practices. Such standards should be adaptable to different healthcare settings, accommodating variations in equipment and patient populations. The ultimate goal is to provide clinicians with a common foundation for conducting oscillometry tests, leading to more accurate and comparable results [59].

Normative data for specific populations: To enhance the clinical utility of oscillometry, further research is imperative to develop normative data specific to various patient populations. Age, gender, and ethnic background significantly influence lung function. Age-related changes, variations in lung size and anatomy based on gender, and differences in genetic factors among ethnic groups can all impact respiratory parameters. Understanding the influence of these demographic characteristics on lung function underscores the importance of having population-specific reference values. This contextualizes the need for accurate diagnosis and assessment, as it ensures that oscillometry results are interpreted within the unique demographic context of each patient. Future directions should prioritize studies that collect comprehensive normative data for diverse patient groups, promoting more accurate and individualized care [20].

Overcoming Technical and Clinical Challenges

Quality assurance: Ensuring the quality and calibration of oscillometry equipment is paramount. Future directions should prioritize implementing robust quality assurance measures to maintain the accuracy and reliability of oscillometry testing equipment. This includes establishing standardized protocols for equipment calibration, regular performance checks, and maintenance routines. A commitment to equipment quality is fundamental to the credibility and consistency of oscillometry results in clinical practice [60].

Interpretation training: Healthcare professionals require specialized training in oscillometry interpretation to derive meaningful insights from the data. Future directions should encompass developing comprehensive educational programs and resources that enhance clinicians' understanding of oscillometry principles, data analysis techniques, and clinical applications. This educational initiative ensures that healthcare providers are proficient in interpreting oscillometry results, reducing the risk of misdiagnosis or mismanagement [61].

Pediatric applications: Specialized research efforts are needed to refine oscillometry techniques for pediatric populations, with particular attention to infants and very young children. Future directions should focus on developing age-appropriate reference values, testing protocols, and equipment modifications to accommodate pediatric patients' unique physiological and behavioral characteristics. By tailoring oscillometry to the specific needs of pediatric populations, its accuracy and effectiveness in diagnosing and monitoring pediatric respiratory conditions can be significantly improved [62].

Incorporating oscillometry into clinical guidelines: Integrating oscillometry into clinical practice guidelines is essential to promote its standardized use and enhance its adoption by healthcare providers. Future directions should involve collaborations between professional societies and guideline development committees, working together to ensure the inclusion of oscillometry in respiratory care recommendations. Accelerating this integration will provide clinicians with clear and authoritative guidance on when and how to utilize oscillometry in patient assessments, ultimately enhancing its clinical impact [63].

Potential for Personalized Medicine

Genetic and molecular profiling: As genetic and molecular profiling technologies advance, integrating these data with oscillometry results holds immense promise. Future directions should explore how genetic markers, such as specific gene variants associated with respiratory conditions, and molecular markers, such as inflammatory biomarkers, can inform treatment decisions. By correlating genetic and molecular profiles with oscillometry data, healthcare providers can tailor treatment strategies to each patient's unique genetic and molecular characteristics, optimizing therapy selection and dosing. This personalized approach can enhance treatment efficacy and minimize adverse effects [64].

AI and machine learning: The burgeoning field of artificial intelligence (AI) and machine learning offers unprecedented opportunities for refining oscillometry's role in personalized medicine. Future directions should focus on developing and refining AI algorithms to analyze oscillometry data alongside genetic, molecular, and clinical information. These algorithms can enhance diagnostic accuracy by recognizing complex patterns and correlations in data. Moreover, AI-driven models have the potential to predict disease progression and provide early warnings of exacerbations, enabling proactive interventions tailored to individual patients. As AI evolves, it will become an indispensable tool in guiding treatment strategies personalized to patients' unique lung function characteristics [65].

Telemedicine and remote monitoring: The expansion of telemedicine and remote monitoring, accelerated by technological advancements, offers the potential for the widespread adoption of oscillometry in home-based care. Future directions should involve the development of user-friendly, home-based oscillometry devices and user interfaces that facilitate remote patient monitoring. Patients can perform oscillometry tests in the comfort of their homes, and the data can be transmitted to healthcare providers in real-time. This approach not only improves accessibility to lung function assessments but also allows for continuous monitoring of patients with chronic respiratory conditions, enabling timely interventions when necessary [66].

Longitudinal studies: Longitudinal studies using oscillometry can provide invaluable insights into disease trajectories and treatment responses. Future research directions should encourage extensive investigations in this area. These studies can elucidate the natural progression of chronic respiratory conditions and identify early predictors of exacerbations or disease worsening. By analyzing longitudinal oscillometry data, healthcare providers can refine and individualize management strategies, adjusting treatments based on each patient's specific needs and response to therapy. This patient-centric approach can improve long-term outcomes and quality of life for individuals with chronic respiratory diseases [67].

Clinical guidelines and recommendations

Inclusion of Oscillometry in Clinical Practice Guidelines

Including oscillometry in clinical practice guidelines is a topic of ongoing development in respiratory medicine. As of the knowledge cutoff, oscillometry has gained recognition for its value in assessing lung function. However, its integration into guidelines must be more consistent across countries and healthcare organizations. To advance its inclusion in future guidelines, several strategies should be considered. Collaboration among experts in respiratory medicine is essential to establishing a robust evidence base for oscillometry's role. Future research should focus on demonstrating its clinical utility in various respiratory conditions, emphasizing early diagnosis, disease monitoring, and personalized treatment. Comparative studies comparing oscillometry with established lung function tests should be conducted to clarify its unique contributions. Moreover, specific guidelines for pediatric respiratory care, addressing age-appropriate reference values and clinical applications, are crucial to ensuring that oscillometry is effectively utilized in children. These efforts are pivotal for realizing oscillometry's full potential in improving lung function assessment accuracy and enhancing patient care in respiratory medicine [11].

Best Practices for Clinicians and Researchers

Education and training: Future guidelines and recommendations must strongly emphasize education and training for healthcare professionals who utilize oscillometry. Comprehensive training programs should be established to equip clinicians with the necessary skills in test administration, data interpretation, and quality assurance. This ensures that healthcare providers are proficient in utilizing oscillometry effectively and obtaining accurate results [68].

Standardized protocols: To guarantee consistency in oscillometry measurements across diverse healthcare settings, developing and adopting standardized testing protocols and reporting templates should be encouraged. These protocols should outline step-by-step procedures for conducting oscillometry tests, including patient preparation, equipment setup, and data collection. Standardization minimizes variability in test administration and interpretation [69].

Quality assurance: Guidelines should provide clear and detailed guidance on quality assurance measures related to oscillometry. These measures should encompass equipment calibration protocols, regular performance checks, and ongoing maintenance practices. Maintaining the accuracy and reliability of oscillometry testing equipment is essential to ensuring that results are consistent and trustworthy [70].

Integration with clinical workflow: Recommendations should address the seamless integration of oscillometry into the clinical workflow. This includes defining the appropriate contexts and circumstances for incorporating oscillometry testing into patient assessments. Guidelines can facilitate its effective implementation in routine clinical practice by guiding when and how to use oscillometry alongside other diagnostic tools [71].

Future Directions for Guideline Development

Global collaboration: In conjunction with regulatory bodies, international collaboration among respiratory and pulmonary medicine societies is pivotal for developing globally recognized guidelines for oscillometry use. Such collaborative efforts can harmonize testing protocols, reference values, and reporting standards, ensuring consistency and facilitating worldwide data exchange among healthcare institutions [72].

Adaptation to emerging technologies: Future guidelines should remain adaptable to the ever-evolving landscape of oscillometry technologies. This includes embracing portable devices, which offer greater accessibility and convenience for clinicians and patients. Furthermore, guidelines should accommodate the integration of remote monitoring capabilities, enabling real-time assessment of lung function and enhancing telehealth services. Incorporating artificial intelligence (AI) and data-driven analysis into guidelines is vital, as these technologies can potentially enhance diagnostic accuracy and predictive capabilities [73].

Patient-centered care: Patient engagement and shared decision-making should be at the forefront of future guidelines. Guidelines should underscore the importance of involving patients in oscillometry discussions, elucidating this technique's benefits and limitations in their care. This collaborative approach empowers patients to participate actively in their healthcare journey and contributes to more informed decisions regarding their lung function assessment and management [74].

Regular updates: Given the rapidly evolving field of respiratory medicine and technology, oscillometry guidelines should be dynamic and subject to regular updates. Staying abreast of new evidence, innovations, and best practices is essential for ensuring clinicians have access to the most current and practical tools for lung function assessment. Regular updates guarantee that guidelines remain relevant in an ever-changing healthcare landscape [75].

Pediatric-specific guidelines: Recognizing oscillometry's unique considerations and applications in pediatric respiratory care, developing pediatric-specific guidelines is imperative. These guidelines should encompass age-appropriate reference values, testing protocols, and clinical applications tailored to the distinct needs of pediatric patients. By addressing these specific requirements, guidelines can enhance the accuracy and effectiveness of oscillometry in pediatric practice, improving the diagnosis and management of respiratory conditions in children [76].

## Conclusions

In conclusion, oscillometry has emerged as a transformative force in modern pulmonary medicine. Its non-invasive, patient-friendly approach offers a comprehensive assessment of lung function, enabling early disease detection, personalized treatment, and improved patient care. While standardization and education challenges persist, oscillometry's potential impact on respiratory medicine is undeniable. As we look to the future, continued research, collaboration, and the integration of emerging technologies promise to elevate oscillometry's role in shaping the landscape of lung function assessment. With its ability to enhance diagnosis, optimize treatment and provide insights into personalized medicine, oscillometry is vital in pursuing better respiratory health and improved patient outcomes.

## References

[1] Ranu H, Wilde M, Madden B (2011). Pulmonary function tests. Ulster Med J.

[2] Gold WM, Koth LL (2015). Pulmonary Function Testing. Murray and Nadel's Textbook of Respiratory Medicine.

[3] Miller MR, Crapo R, Hankinson J (2005). General considerations for lung function testing. Eur Respir J.

[4] Ponce MC, Sankari A, Sharma S (2023). Pulmonary Function Tests. https://pubmed.ncbi.nlm.nih.gov/29493964/.

[5] Komarow HD, Myles IA, Uzzaman A, Metcalfe DD (2011). Impulse oscillometry in the evaluation of diseases of the airways in children. Ann Allergy Asthma Immunol.

[6] Lundblad LK, Robichaud A (2021). Oscillometry of the respiratory system: a translational opportunity not to be missed. Am J Physiol Lung Cell Mol Physiol.

[7] Walker HK (1990). The Origins of the History and Physical Examination. Clinical Methods: The History, Physical, and Laboratory Examinations. 3rd edition.

[8] Vatanoğlu-Lutz EE, Ataman AD (2016). Medicine in philately: Rene T. H. Laënnec, the father of stethoscope. Anatol J Cardiol.

[9] Lamb K, Theodore D, Bhutta BS (2023). Spirometry. https://pubmed.ncbi.nlm.nih.gov/32809361/.

[10] Porojan-Suppini N, Fira-Mladinescu O, Marc M, Tudorache E, Oancea C (2020). Lung function assessment by impulse oscillometry in adults. Ther Clin Risk Manag.

[11] Liang X, Zheng J, Gao Y (2022). Clinical application of oscillometry in respiratory diseases: an impulse oscillometry registry. ERJ Open Res.

[12] Desiraju K, Agrawal A (2016). Impulse oscillometry: The state-of-art for lung function testing. Lung India.

[13] Bhattarai P, Myers S, Chia C, Weber HC, Young S, Williams AD, Sohal SS (2020). Clinical application of forced oscillation technique (FOT) in early detection of airway changes in smokers. J Clin Med.

[14] Pan J, Saltos A, Smith D, Johnson A, Vossoughi J (2013). Comparison of respiratory resistance measurements made with an airflow perturbation device with those from impulse oscillometry. J Med Eng.

[15] Lu L, Peng J, Wu F (2023). Clinical characteristics of airway impairment assessed by impulse oscillometry in patients with chronic obstructive pulmonary disease: findings from the ECOPD study in China. BMC Pulm Med.

[16] Kaczka DW, Dellacá RL (2011). Oscillation mechanics of the respiratory system: applications to lung disease. Crit Rev Biomed Eng.

[17] Brashier B, Salvi S (2015). Measuring lung function using sound waves: role of the forced oscillation technique and impulse oscillometry system. Breathe (Sheff).

[18] Navajas D, Farré R (2001). Forced oscillation assessment of respiratory mechanics in ventilated patients. Crit Care.

[19] Bhatawadekar SA, Leary D, de Lange V (2019). Reactance and elastance as measures of small airways response to bronchodilator in asthma. J Appl Physiol (1985).

[20] Berger KI, Wohlleber M, Goldring RM (2021). Respiratory impedance measured using impulse oscillometry in a healthy urban population. ERJ Open Res.

[21] Campbell M, Sapra A (2023). Physiology, Airflow Resistance. https://pubmed.ncbi.nlm.nih.gov/32119288/.

[22] Milne S, Jetmalani K, Chapman DG, Duncan JM, Farah CS, Thamrin C, King GG (2019). Respiratory system reactance reflects communicating lung volume in chronic obstructive pulmonary disease. J Appl Physiol (1985).

[23] King GG, Bates J, Berger KI (2020). Technical standards for respiratory oscillometry. Eur Respir J.

[24] Bednarek M, Grabicki M, Piorunek T, Batura-Gabryel H (2020). "Current place of impulse oscillometry in the assessment of pulmonary diseases.". Respir Med.

[25] Hoogsteden HC, Verhoeven GT, Lambrecht BN, Prins JB (1999). Airway inflammation in asthma and chronic obstructive pulmonary disease with special emphasis on the antigen-presenting dendritic cell: influence of treatment with fluticasone propionate. Clin Exp Allergy.

[26] Kakavas S, Kotsiou OS, Perlikos F, Mermiri M, Mavrovounis G, Gourgoulianis K, Pantazopoulos I (2021). Pulmonary function testing in COPD: looking beyond the curtain of FEV1. NPJ Prim Care Respir Med.

[27] Mueller-Mang C, Ringl H, Herold C (2017). Interstitial lung diseases. Multislice CT.

[28] Deepak D, Prasad A, Atwal SS, Agarwal K (2017). Recognition of small airways obstruction in asthma and COPD - the road less travelled. J Clin Diagn Res.

[29] Rodriguez E, Bullard CM, Armani MH, Miller TL, Shaffer TH (2013). Comparison study of airway reactivity outcomes due to a pharmacologic challenge test: impulse oscillometry versus least mean squared analysis techniques. Pulm Med.

[30] Loukou I, Moustaki M, Deligianni A, Sardeli O, Douros K (2021). Forced oscillation technique for monitoring the respiratory status of children with cystic fibrosis: a systematic review. Children (Basel).

[31] Rao L, Tiller C, Coates C (2010). Lung growth in infants and toddlers assessed by multi-slice computed tomography. Acad Radiol.

[32] Nucera E, Rizzi A, Agrosì C, Lohmeyer FM, Inchingolo R (2022). Lung function tests, quality of life and telemedicine: three windows on the multifaceted world of asthma in adolescents. Children (Basel).

[33] Molino A, Simioli F, Stanziola AA, Mormile M, Martino M, D'Amato M (2017). Effects of combination therapy indacaterol/glycopyrronium versus tiotropium on moderate to severe COPD: evaluation of impulse oscillometry and exacerbation rate. Multidiscip Respir Med.

[34] Saadeh C, Saadeh C, Cross B, Gaylor M, Griffith M (2015). Advantage of impulse oscillometry over spirometry to diagnose chronic obstructive pulmonary disease and monitor pulmonary responses to bronchodilators: An observational study. SAGE Open Med.

[35] Gupta N, Sachdev A, Gupta D, Gupta S (2021). Oscillometry - The future of estimating pulmonary functions. KPJ.

[36] Wei X, Shi Z, Cui Y (2017). Impulse oscillometry system as an alternative diagnostic method for chronic obstructive pulmonary disease. Medicine (Baltimore).

[37] Jung T, Vij N (2021). Early diagnosis and real-time monitoring of regional lung function changes to prevent chronic obstructive pulmonary disease progression to severe emphysema. J Clin Med.

[38] de Oliveira Jorge PP, de Lima JH, Chong E Silva DC, Medeiros D, Solé D, Wandalsen GF (2019). Impulse oscillometry in the assessment of children's lung function. Allergol Immunopathol (Madr).

[39] Dandurand RJ, Lavoie JP, Lands LC, Hantos Z (2019). Comparison of oscillometry devices using active mechanical test loads. ERJ Open Res.

[40] Veldhoen ES, Roos JH, Bekkema R (2022). Oscillometry: A substitute of spirometry in children with neuromuscular diseases?. Pediatr Pulmonol.

[41] Staff E (2023). Instrumentation tools: Calibration of measuring instruments: significance, costs & risks. https://instrumentationtools.com/calibration-of-measuring-instruments/.

[42] Lundblad LK, Miletic R, Piitulainen E, Wollmer P (2019). Oscillometry in chronic obstructive lung disease: In vitro and in vivo evaluation of the impulse oscillometry and tremoflo devices. Sci Rep.

[43] Park JH, Lee JH, Kim HJ, Jeong N, Jang HJ, Kim HK, Park CS (2019). Usefulness of impulse oscillometry for the assessment of bronchodilator response in elderly patients with chronic obstructive airway disease. J Thorac Dis.

[44] Muntner P, Einhorn PT, Cushman WC (2019). Blood pressure assessment in adults in clinical practice and clinic-based research: JACC scientific expert panel. J Am Coll Cardiol.

[45] Mendez I, Van den Hof MC (2013). Mobile remote-presence devices for point-of-care health care delivery. CMAJ.

[46] Kostorz-Nosal S, Jastrzębski D, Błach A, Skoczyński S (2023). Window of opportunity for respiratory oscillometry: A review of recent research. Respir Physiol Neurobiol.

[47] A A, Dahan F, Alroobaea R (2023). A smart IoMT based architecture for E-healthcare patient monitoring system using artificial intelligence algorithms. Front Physiol.

[48] Makan G, Dandurand RJ, Gingl Z, Hantos Z (2022). Intra-breath changes in respiratory mechanics assessed from multi-frequency oscillometry measurements. Physiol Meas.

[49] Lee HJ, Kim HS, Yoon JS (2023). Impulse oscillometry system for assessing small airway dysfunction in pediatric bronchiolitis obliterans; association with conventional pulmonary function tests. PLoS One.

[50] Hsia CC, Bates JH, Driehuys B (2023). Quantitative imaging metrics for the assessment of pulmonary pathophysiology: an official American Thoracic Society and Fleischner Society joint workshop report. Ann Am Thorac Soc.

[51] Tzouvelekis A, Kouliatsis G, Anevlavis S, Bouros D (2005). Serum biomarkers in interstitial lung diseases. Respir Res.

[52] Davenport T, Kalakota R (2019). The potential for artificial intelligence in healthcare. Future Healthc J.

[53] Streeter OE Jr, Beron PJ, Iyer PN (2017). Precision medicine: genomic profiles to individualize therapy. Otolaryngol Clin North Am.

[54] (2020). Prevalence and attributable health burden of chronic respiratory diseases, 1990-2017: a systematic analysis for the Global Burden of Disease Study 2017. Lancet Respir Med.

[55] Kumar Y, Koul A, Singla R, Ijaz MF (2023). Artificial intelligence in disease diagnosis: a systematic literature review, synthesizing framework and future research agenda. J Ambient Intell Humaniz Comput.

[56] Congrete S, Metersky ML (2021). Telemedicine and remote monitoring as an adjunct to medical management of bronchiectasis. Life (Basel).

[57] Larsen GL, Morgan W, Heldt GP (2009). Impulse oscillometry versus spirometry in a long-term study of controller therapy for pediatric asthma. J Allergy Clin Immunol.

[58] Kouri A, Dandurand RJ, Usmani OS, Chow CW (2021). Exploring the 175-year history of spirometry and the vital lessons it can teach us today. Eur Respir Rev.

[59] Deprato A, Ferrara G, Bhutani M (2022). Reference equations for oscillometry and their differences among populations: a systematic scoping review. Eur Respir Rev.

[60] Wu JK, DeHaas E, Nadj R (2020). Development of quality assurance and quality control guidelines for respiratory oscillometry in clinic studies. Respir Care.

[61] Cross AJ, Thomas D, Liang J, Abramson MJ, George J, Zairina E (2022). Educational interventions for health professionals managing chronic obstructive pulmonary disease in primary care. Cochrane Database Syst Rev.

[62] Frei J, Jutla J, Kramer G, Hatzakis GE, Ducharme FM, Davis GM (2005). Impulse oscillometry: reference values in children 100 to 150 cm in height and 3 to 10 years of age. Chest.

[63] Institute of Medicine (US) Committee on Standards for Developing Trustworthy Clinical Practice Guidelines (2011). Promoting Adoption of Clinical Practice Guidelines. Clinical Practice Guidelines We Can Trust. National Academies Press.

[64] Sridhar K, Singh A, Butzmann A, Jangam D, Ohgami RS (2019). Molecular genetic testing methodologies in hematopoietic diseases: current and future methods. Int J Lab Hematol.

[65] Schork NJ (2019). Artificial intelligence and personalized medicine. Cancer Treat Res.

[66] Haleem A, Javaid M, Singh RP, Suman R (2021). Telemedicine for healthcare: capabilities, features, barriers, and applications. Sens Int.

[67] Fuchs O, Bahmer T, Weckmann M (2018). The all age asthma cohort (ALLIANCE) - from early beginnings to chronic disease: a longitudinal cohort study. BMC Pulm Med.

[68] Worsley C, Webb S, Vaux E (2016). Training healthcare professionals in quality improvement. Future Hosp J.

[69] Yamamoto A, Shirai T, Hirai K (2020). Oscillometry as a predictor of exercise tolerance in COPD. COPD.

[70] Cao X, Song C, Guo L (2015). Quality control and validation of oscillometric blood pressure measurements taken during an epidemiological investigation. Medicine (Baltimore).

[71] Tu SW, Musen MA, Shankar R (2004). Modeling guidelines for integration into clinical workflow. Stud Health Technol Inform.

[72] Rutter M, Camillo CA, Coss P (2019). European Respiratory Society International Congress 2018: Allied Respiratory Professionals' report of highlighted sessions. ERJ Open Res.

[73] Liao Y, Thompson C, Peterson S, Mandrola J, Beg MS (2019). The future of wearable technologies and remote monitoring in health care. Am Soc Clin Oncol Educ Book.

[74] Armstrong MJ, Shulman LM, Vandigo J, Mullins CD (2016). Patient engagement and shared decision-making: What do they look like in neurology practice?. Neurol Clin Pract.

[75] Lundblad LKA, Siddiqui S, Bossé Y, Dandurand RJ (2021). Applications of oscillometry in clinical research and practice. Can J Respir Crit Care Sleep Med.

[76] (2015). Pediatric acute respiratory distress syndrome: consensus recommendations from the Pediatric Acute Lung Injury Consensus Conference. Pediatr Crit Care Med.

